# Supplementary material for: Wavepacket insights into the photoprotection mechanism of the UV filter methyl anthranilate
Source: Nat Commun. 2018 Dec 5;9:5188. doi: 10.1038/s41467-018-07681-1 (PMC6281654; doi:10.1038/s41467-018-07681-1)
Supplement: Supplementary file 1 — Supplementary Information [file 41467_2018_7681_MOESM1_ESM.pdf]

# Supplementary Information

## Wavepacket insights into the photoprotection mechanism of the UV filter methyl anthranilate

Rodrigues *et al.*

# Supplementary Data 1

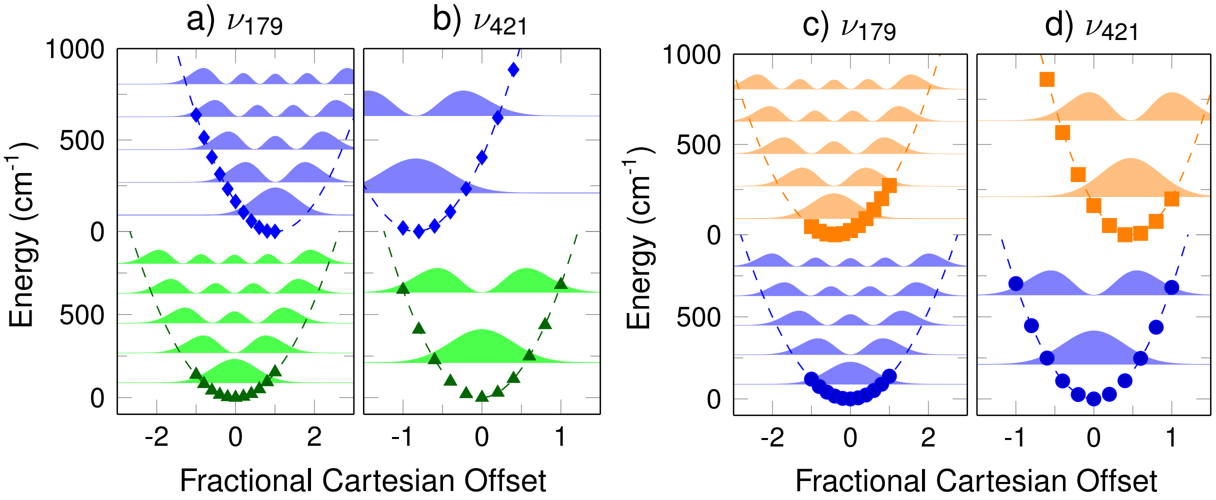


**Supplementary Figure 1:** Potential energy cuts along the 179 cm$^{-1}$ (a and c) and 421 cm$^{-1}$ (b and d) normal mode coordinates on the $S_{0,eq}$ (green triangles), $S_{1,vFC}$ (blue diamonds), $S_{1,eq}$ (blue circles), and $D^{+}_{0,vFC}$ (orange squares) surfaces. Quadratic fits to the calculated points are shown as dashed lines of the corresponding colour. Positive Cartesian offset is shown by the vector displacements in Figure 2 in the main paper. Probability densities of the solutions to a quantum harmonic oscillator are shown for each vibrational level.

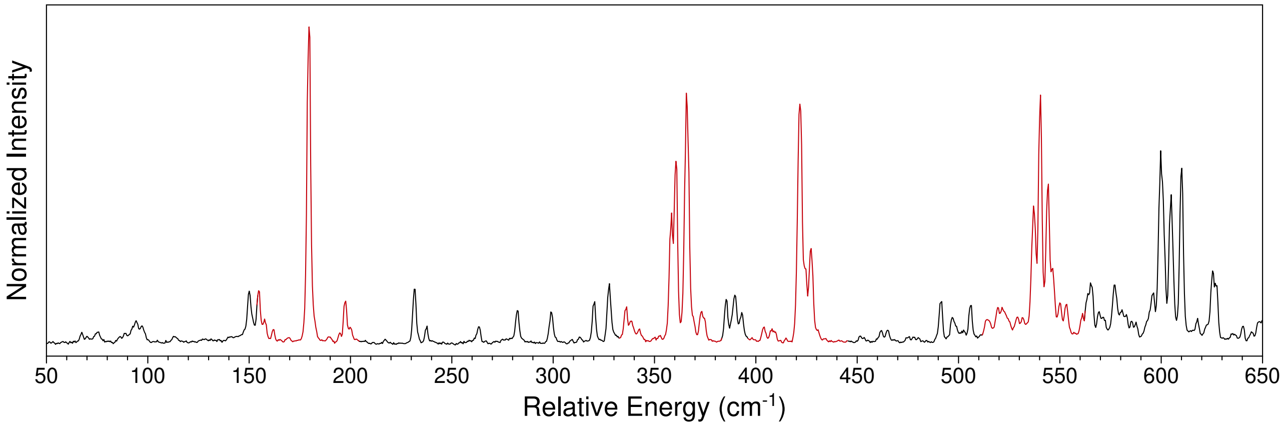


**Supplementary Figure 2:** Enlarged LIF spectrum showing the satellite features within 25 cm$^{-1}$ (red line) of each $\nu_{179}$ and $\nu_{421}$ level.

**Supplementary Table 1:** Low-lying vibrational frequencies (in $cm^{-1}$) calculated at the $S_{0,eq}$, $S_{1,eq}$, and $D^{+}_{0,eq}$ equilibrium geometries of MA.

| $S_{0,eq}$ | $S_{1,eq}$ | $D^{+}_{0,eq}$ | $S_1$ Expt. |
|---|---|---|---|
| 51 | 80 | 75 | |
| 75 | 88 | 120 | |
| 127 | 124 | 155 | |
| 157 | 160 | 167 | |
| **183** | **185** | **180** | **179** |
| 196 | 223 | 248 | |
| 261 | 331 | 331 | |
| 342 | 339 | 361 | |
| **364** | **378** | **417** | **366** |
| **421** | **431** | **419** | **421** |

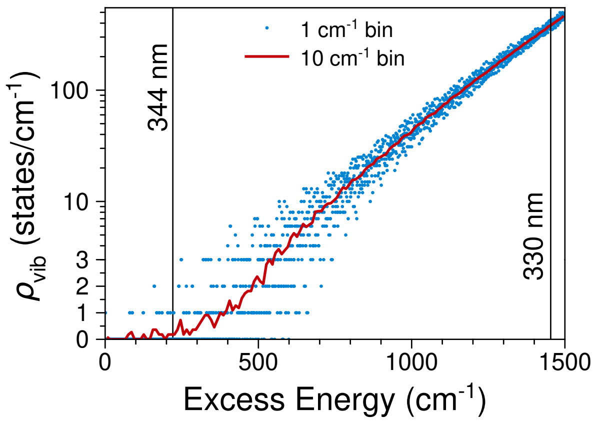


**Supplementary Figure 3:** Density of $S_1$ vibrational states calculated using the $S_{1,eq}$ harmonic frequencies and the Beyer-Swinehart algorithm[1] for a 1 $cm^{-1}$ energy bin (blue dots) and the average density in a 10 $cm^{-1}$ bin (red line). Energy is relative to the vibronic origin of the $S_1$ state.

**Supplementary Table 2:** Converged fit parameters from Eqs. (S3) or (S4) for the one-dimensional transients of MA and MenA at all pump wavelengths.

| Molecule | $\lambda_{pu}$ (nm) | Beat Frequency ($cm^{-1}$) | Amplitude (arb. unit) | Phase |
|---|---|---|---|---|
| MA | 348 | 178 | 0.095 | 0.39 $\pi$ |
| | | 5 | 0.15 | 0.51 $\pi$ |
| MA | 344 | 243 | 0.95 | 0 $\pi$ |
| | | 182 | 2.34 | 0.32 $\pi$ |
| | | 115 | −1.54 | 0.08 $\pi$ |
| | | 63 | 2.63 | 0 $\pi$ |
| MA | 330 | 178 | 0.21 | 0.34 $\pi$ |
| | | 63 | 0.07 | 0 $\pi$ |
| MenA | 330 | 159 | 0.25 | 0.40 $\pi$ |
| | | 77 | 0.26 | 0.34 $\pi$ |

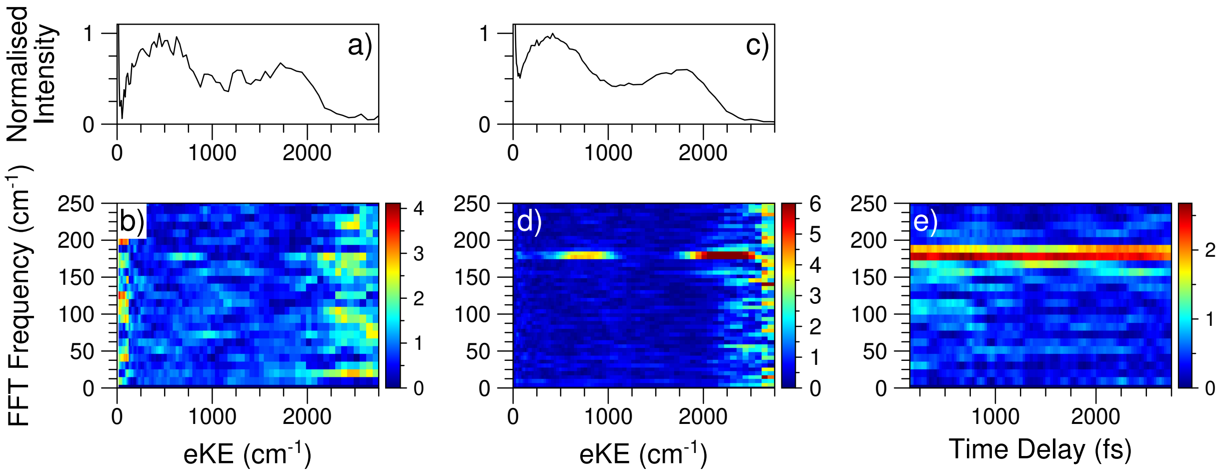


**Supplementary Figure 4:** Example photoelectron spectra and FFT(eKE) for $\lambda_{pu} = 351$ nm (a and b) and 348 nm (c and d). e) FFT($\Delta t$) at $\lambda_{pu} = 348$ nm for eKE $\approx$ 1800–2600 $cm^{-1}$. As the two TR-PES features at each $\lambda_{pu}$ have the same temporal behavior, the photoelectron spectra shown in a and c were averaged over several $\Delta t$ for an improved signal-to-noise ratio.

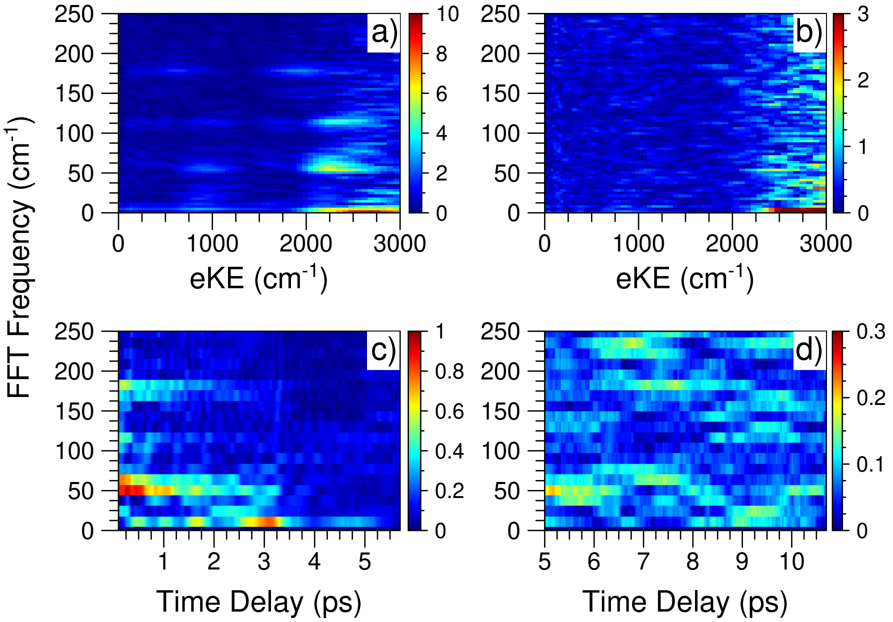


**Supplementary Figure 5:** FFT(eKE) for $\lambda_{pu}$ = 330 nm from a) $\Delta t$ = 0−7.5 ps and b) 5−12.5 ps. FFT($\Delta t$) for the same time-delay windows are shown in c) and d), respectively.

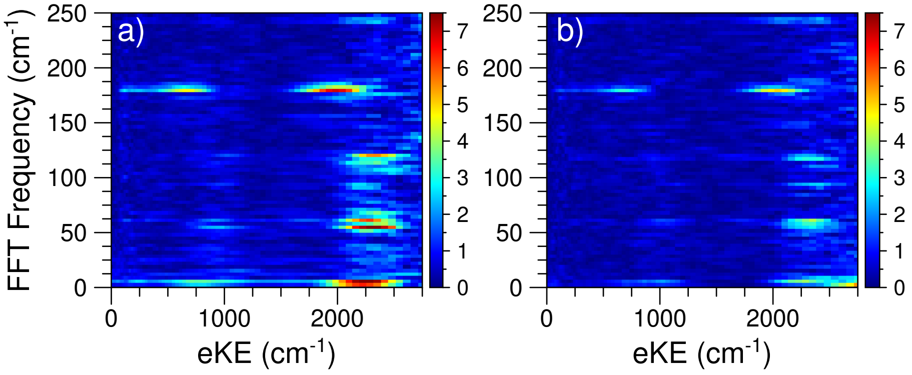


**Supplementary Figure 6:** FFT(eKE) spectra from MA taken at $\lambda_{pu}$ = 344 nm for (a) $\Delta t$ = 0−7.5 ps and (b) $\Delta t$ = 5−12.5 ps.

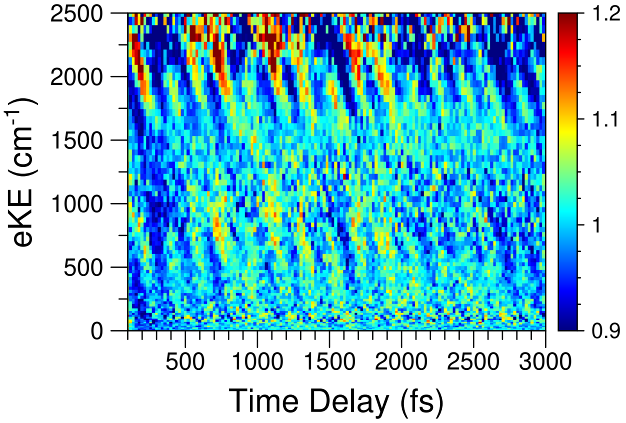


**Supplementary Figure 7:** Portion of a "flattened" two-dimensional TR-PES transient of MA at $\lambda_{\text{pu}} = 344$ nm. The color map intensity has been selected for visual clarity of the signal oscillations. The diagonal nature of the recurrances show that the location of the Franck-Condon ionization window is not shared between all vibrational modes.

# Supplementary Discussion 1

Photoelectron spectra with a double peak are common for molecules in which excited state proton transfer, akin to a *keto-enol* tautomerization, may occur.[2] However, given that excited state proton or H-atom transfer in MA does not yield a stable tautomer (*i.e.* there is only one excited state minimum geometry along the tautomerization coordinate) and the two TR-PES features have the same temporal behavior,[3] it is unlikely that the two peaks observed in the photoelectron spectra are due to different tautomers. Furthermore, the photoelectron spectrum at $\lambda_{\text{pu}} = 351$ nm, shown in Supplementary Figure 4, also shows two peaks; as this pump wavelength is centered below the $S_1 \leftarrow S_0$ origin, the excess energy shown in the photoelectron structure must be from the probe step. Thus, the double-peak photoelectron spectra of MA are consistent with different couplings between the $S_1$ state and the corresponding vibrational modes of the $D_0^+$ state. The high eKE feature is primarily due to ionization to the $D_0^+$ (v = 0), with some vibrational excitation retained in the cationic $\nu_{179}$ (as the 180 cm$^{-1}$ beat is observed at slightly lower eKE; see Supplementary Figures 5 and 6). The low eKE feature corresponds to the first vibrational excited level of a cationic mode with $\Delta E_{\text{vib}} \approx 1400$ cm$^{-1}$; the high eKE edge of which corresponds to the v = 0 levels of the other cationic modes as evidenced by the reappearance of the same quantum beats. Indeed, the previously reported PES with a shorter probe wavelength show a similar progression with the same energy difference,[3] supporting the conclusion that the PES structure is due to vibrational excitation of a cationic mode. Normal mode calculations of the MA cation reveal several modes around 1400 cm$^{-1}$ whose motions may be generally described as a combination of ring breathing and stretches of the amine and/or carbonyl groups.

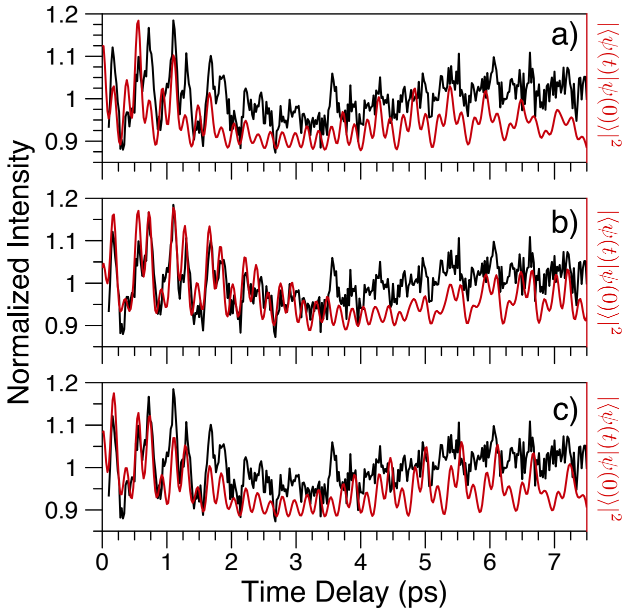


**Supplementary Figure 8:** Example 344 nm transient (black line) and time-autocorrelation (red line) for a) $\phi_n = 0$ for all $n$, b) $\phi_n = \pi/2$ as specified in Supplementary Table 3 in the Supplementary Methods, and c) $\phi_n = \pi/2$ for all $n$ contributing to a 180 cm$^{-1}$ quantum beat.

# Supplementary Discussion 2

In order to match the $\lambda_{\text{pu}} = 344$ nm transient shown in the main paper and experimentally determined beat phases shown in Supplementary Table 2, it was necessary to add a phase factor ($\phi_n$) to the wavepacket calculation as described in the Supplementary Methods. As seen in Supplementary Figure 8 a, an absence of any phase information results in a wavepacket simulation that does not reproduce the changes in beat intensity. Both Supplementary Figure 8 b and c reproduce the experimental 180 cm$^{-1}$ beat phase of $\sim \pi/2$ relative to the other beats, however the best agreement (Supplementary Figure 8 b) is achieved when some of the 180 cm$^{-1}$ beat sources, namely the LIF peak at 366 cm$^{-1}$ and the cluster of peaks near 600 cm$^{-1}$, do not have the same phase. By keeping the phase factors of the 366 and ~600 cm$^{-1}$ vibrational modes different than for $\nu_{179}$, the amplitude and timing of the 180 cm$^{-1}$ beat revivals are in better agreement with experiment. These results thus support the conclusions that the 366 cm$^{-1}$ LIF peak is a separate vibrational mode (see Supplementary Table 1) and that the experimental beat phase is due to a vibrational mode specific ionization window.

# Supplementary Methods

## Kinetic Models

### Instrument Response Function

The instrument response function (IRF) was taken to be the time resolved ion yield from the $2+1$ non-resonant ionization of Xe. These transients were fit to a Gaussian function to measure the cross-correlation of the two laser pulses in Eqs. (S1)–(S4). These fits were taken to be the full width at half maximum (FWHM) of the Gaussian fit; $100-120$ fs were typical.

### Overall Decay Model

The model used to fit our 1-D transient data assume parallel excited state decay pathways, *i.e.* all dynamics begin at $\Delta t = 0$. Briefly, a sum of exponential decay functions are multiplied by a step function and then convoluted with a Gaussian function to model the IRF. The total model is thus:

$$S(t) = S_0 + g(t) * \left[ H(t) \sum_i A_i \exp\left(-\frac{t}{\tau_i}\right) \right] \tag{S1}$$

where $t = \Delta t - t_0$, $S(t)$ is the time delay-dependent signal, $S_0$ is the signal baseline, $g(t)$ is the Gaussian function, $A_i$ are the amplitudes, and $\tau_i$ the time constants. As the overall dynamics for MA are quite slow, the maximum temporal overlap at $t_0$ (*i.e.* $\Delta t = 0$) was assumed to be located halfway up the initial rise in TR-PES signal. $H(t)$ is a step function such that:

$$H(t) = \begin{cases} 0 & \text{if } t < 0, \\ 1 & \text{if } t \geq 0. \end{cases}$$

Similarly, our 2-D transient data (*i.e.* eKE *vs.* $\Delta t$) were fit using global lifetime analysis[4,5] and the following functional form:

$$S(t, \text{eKE}) = g(t) * \left[ H(t) \sum_i \text{DAS}_i(\text{eKE}) \cdot \exp\left(-\frac{t}{\tau_i}\right) \right] \tag{S2}$$

where $\text{DAS}_i(\text{eKE})$ are the "decay associated spectra:" eKE-dependent exponential decay amplitudes for a given time constant.[4]

### Beat Frequencies

For 1-D transients with observable beat frequencies, sine functions were added to Eq. (S1) such that the total model becomes:

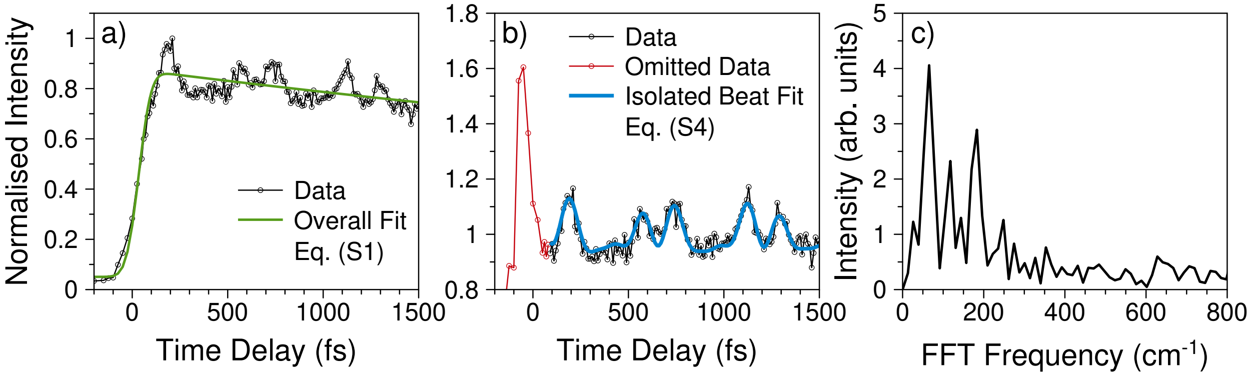


**Supplementary Figure 9:** a) Example 1-D transient at $\lambda_{\mathrm{pu}} = 344$ nm with a fit using Eq. (S1). b) The resulting isolated transient along with a fit using Eq. (S4). Data shown in red were omitted from the fit and FFT analysis due to the distortion near $\Delta t = 0$. c) 1-D FFT spectrum from an isolated transient at $\lambda_{\mathrm{pu}} = 344$ nm.

$$S_{\mathrm{beat}}(t) = S(t) \times B \exp\left(-\frac{t}{\tau_{\mathrm{beats}}}\right) \sum_j A_j \sin(\omega_j \cdot t + \phi_j) \tag{S3}$$

where $\tau_{\mathrm{beats}}$ is the dampening lifetime of the total beat signal with amplitude $B$, $A_j$ are the amplitudes of each frequency $\omega_j$, and $\phi_j$ are phase shifts. As discussed below, isolated transients that have been normalized by the fit from Eq. (S1) are fit using the dampened sine functions of Eq. (S3) with an additive baseline offset ($S_0$):

$$S_{\mathrm{flat}}(t) = S_0 + B \exp\left(-\frac{t}{\tau_{\mathrm{beats}}}\right) \sum_j A_j \sin(\omega_j \cdot t + \phi_j). \tag{S4}$$

As an alternative to Eqs. (S3) and (S4), the beat dampening time $\tau_{\mathrm{beats}}$ could reasonably be beat-dependent (*i.e.* indexed by $j$). Indeed, as seen in Figure 3 in the main paper, the different beat frequencies appear to dampen with different lifetimes. However, based on the results at $\lambda_{\mathrm{pu}} = 344$ nm and given the difficulty in differentiating between dephasing and IVR, the single "global" beat decay time was chosen.

## Transient Fitting and Fourier Transform Analysis

### Overall Processing

To isolate the temporal behavior of the quantum beats, transients were first fit using Eqs. (S1) or (S2). The transient signal was then "normalized" by dividing the signal by the model fit, resulting in a "flattened" transient (see Supplementary Figure 9). Thus, decreases in quantum beat intensity due to decreased total signal intensity are removed and any remaining temporal behavior may be attributed to dynamics associated with the quantum beats. These "isolated" transients were then either fit with (S4) and/or prepared for fast Fourier transform

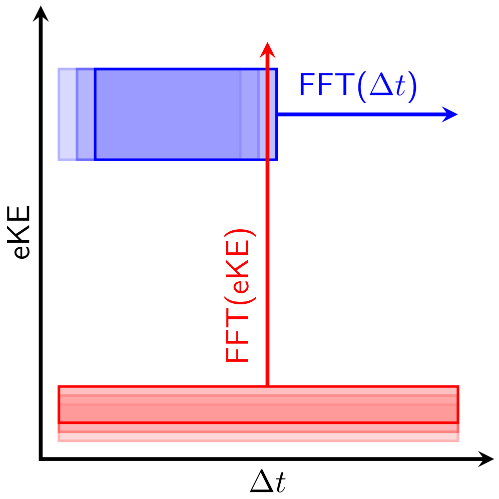


**Supplementary Figure 10:** Schematic representation of the production of FFT(eKE) and FFT($\Delta t$).

(FFT) analysis as described in Ref. [6]. To minimize interference from the rise in signal at $\Delta t = 0$, all isolated transient fits and FFT analyses were performed on transient signal from $\Delta t > 0$; Supplementary Figure 9 b shows the time delays omitted from the analysis.

### FFT(eKE) and FFT($\Delta t$) Spectra

In addition to the 1-D FFT spectra generated from the total photoelectron signal (*i.e.* all eKE and $\Delta t$), FFT spectra were also generated as functions of eKE and $\Delta t$, as shown in the main paper and Supplementary Figures 4, 5, and 6. The former were produced by averaging the photoelectron signal in a fixed window in photoelectron velocity ($v_{e^-}$), then performing an FFT analysis on this average signal for all $\Delta t$. The window is then shifted by one velocity bin and a new FFT spectrum generated. This "sliding window" procedure is repeated until the high velocity edge of the window reaches the maximum $v_{e^-}$. The resulting FFT($v_{e^-}$) are then transformed into FFT(eKE) using the average eKE of each velocity window. Window size was subjectively chosen to maximize the FFT signal-to-noise ratio while maintaining eKE resolution.

Similarly, FFT($\Delta t$) were produced by first averaging the isolated 2-D transient over a given eKE. FFT analysis was then performed on the 1-D transient over a fixed $\Delta t$ window. The window was then advanced one time delay (*i.e.* the step size of the laser delay stage) and the FFT analysis repeated until the high $\Delta t$ edge of the window reached the maximum time delay, similar to the procedure described in Ref. [7]. The final $\Delta t$ values presented are the first time delay in each window. Window size was subjectively chosen to be the smallest number of time delays that reproduced an FFT spectrum with the observed quantum beat frequencies. The production of both FFT(eKE) and FFT($\Delta t$) are shown schematically in Supplementary Figure 10.

## Wavepacket Calculation

To help interpret the observed beat behavior, the time-autocorrelation function of the photoexcited wavepacket $\langle\psi(t)|\psi(0)\rangle$ was calculated at $\lambda_{\text{pu}} = 348$ and 344 nm. Rewriting Eq. (1) from the main paper, the time-autocorrelation function is:[8]

$$\langle\psi(t)|\psi(0)\rangle = \sum_n a_n^2\, g_n^2 \exp\left[-iE_n t/\hbar\right], \tag{S5}$$

$$= \sum_n a_n^2\, g_n^2 \cos\left[E_n t/\hbar\right] - i\, a_n^2\, g_n^2 \sin\left[E_n t/\hbar\right], \tag{S6}$$

where $t$ is the wavepacket propagation time, $a_n^2$ is the Franck-Condon factor for each $S_1(\text{v} = n) \leftarrow S_0(\text{v} = 0)$ transition, and $E_n$ is the energy of level $n$ of the $S_1$ state. The $a_n^2$ and $E_n$ were taken from the most prominent features of the LIF spectrum shown in the main paper (see Supplementary Table 3), though we recognize that the LIF amplitudes will not be equivalent to the Franck-Condon factors for the initial photoexcitation. Laser excitation is described by $g_n^2 = \exp[-4\ln 2(E_n - \bar{E})^2/\Delta E^2]$, where $\bar{E}$ and $\Delta E$ are the average energy and FWHM of the pump laser pulse, respectively. For each wavelength, $\bar{E}$ and $\Delta E$ were determined by fitting the pump laser spectrum shown in Figure 2 of the main paper using a Gaussian function; pump laser characteristics are given in Supplementary Table 4. From fits to the transients using Eqs. (S3) or (S4), the 180 cm$^{-1}$ beat has phase of approximately $\pi/2$ relative to the other beats. As such, a phase shift was added to Eq. (S6) resulting in:

$$\langle\psi(t)|\psi(0)\rangle = \sum_n a_n^2\, g_n^2 \cos\left[E_n t/\hbar + \phi_n\right] - i\, a_n^2\, g_n^2 \sin\left[E_n t/\hbar + \phi_n\right] \tag{S7}$$

where $\phi_n$ is the relative phase of the vibrational eigenstate at the Franck-Condon region. For direct comparison to the experimental transient, the absolute square of Eq. (S7) (*i.e.* $|\langle\psi(t)|\psi(0)\rangle|^2$) may be evaluated, yielding:

$$|\langle\psi(t)|\psi(0)\rangle|^2 = \sum_{n=1} |a_n^2 g_n^2|^2 + \sum_{n=2}\sum_{m<n} |a_n^2 g_n^2||a_m^2 g_m^2| \cos\left[\frac{(E_n - E_m)t}{\hbar} + (\phi_n - \phi_m)\right]. \tag{S8}$$

Equation (S8) is in agreement with the beat equation given by Stolow, Bragg, and Neumark[9] assuming that the transition to the single, final state has unit probability. The absolute square of Eq. (S7) was numerically evaluated using the parameters in Supplementary Table 3 and experimental time steps for a total duration of $\leq$12.5 ps; a plot of $|\langle\psi(t)|\psi(0)\rangle|^2$ versus $\Delta t$ at $\lambda_{\text{pu}} = 344$ nm is shown in Supplementary Figure 11 a along with the corresponding FFT spectrum (as described above) in Supplementary Figure 11 b.

To qualitatively account for the temporal widths of our pump and probe laser pulses, the numeric results of $|\langle\psi(t)|\psi(0)\rangle|^2$ were "blurred" by the IRF according to the following procedure. Assuming Gaussian time profiles, the temporal width of each laser pulse was determined from an IRF measurement (see above), yielding a FWHM of $\sim$73 fs for each pulse. The time-autocorrelation amplitude at each $t$ was then replaced by a Gaussian of equal area with FWHM = 73 fs. The Gaussian amplitudes at each time delay are then summed,

**Supplementary Table 3:** LIF data and relative phase used for the wavepacket calculations described in the Supplementary Methods.

| Peak Energy ($cm^{-1}$) | LIF Intensity (arb. unit) | Relative Phase |
|---|---|---|
| 0 | 1 | 0 |
| 179.23955 | 0.60926 | $\pi/2$ |
| 358.16028 | 0.26491 | $\pi/2$ |
| 360.29342 | 0.36068 | $\pi/2$ |
| 365.41422 | 0.48671 | 0 |
| 421.43324 | 0.46646 | 0 |
| 427.00410 | 0.19912 | 0 |
| 536.70895 | 0.27791 | $\pi/2$ |
| 540.16398 | 0.48318 | $\pi/2$ |
| 544.05185 | 0.31840 | $\pi/2$ |
| 546.21222 | 0.16242 | $\pi/2$ |
| 599.45758 | 0.38003 | 0 |
| 604.66258 | 0.29875 | 0 |
| 609.86942 | 0.34782 | 0 |
| 714.39411 | 0.12081 | $\pi/2$ |
| 718.76540 | 0.24157 | $\pi/2$ |
| 725.76216 | 0.24652 | 0 |
| 728.38680 | 0.22270 | 0 |
| 773.07703 | 0.24103 | 0 |
| 784.92941 | 0.45394 | 0 |
| 790.20017 | 0.35175 | 0 |
| 840.36614 | 0.12820 | 0 |
| 844.77477 | 0.28428 | 0 |
| 850.94904 | 0.11625 | 0 |

**Supplementary Table 4:** Pump laser spectral characteristics used in the wavepacket calculations described in the Supplementary Methods.

| $\lambda_{pu}$ | Central Energy ($cm^{-1}$) | FWHM ($cm^{-1}$) |
|---|---|---|
| 351 | 28471 | 578 |
| 348 | 28750 | 598 |
| 344 | 29099 | 540 |
| 330 | 30288 | 551 |

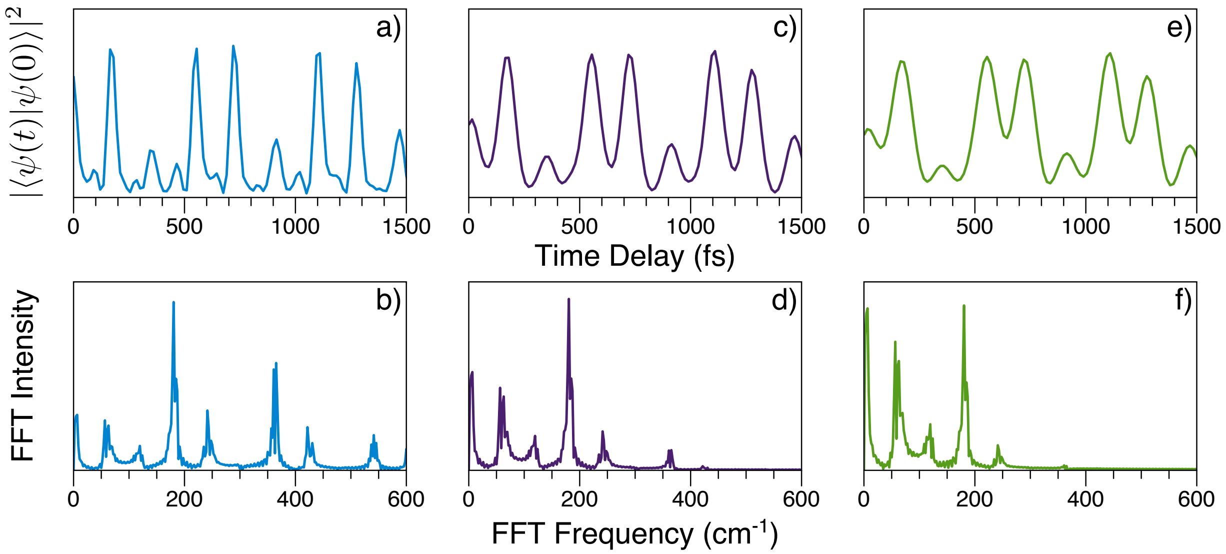


**Supplementary Figure 11:** Time-autocorrelation "transients" and 1-D FFT spectra at $\lambda_{\rm pu}$ = 344 nm showing the "raw" results in blue (a and b), pump "blurred" results in purple (c and d), and the pump-probe "blurred" results in green (e and f).

yielding the pump "blurred" autocorrelation function shown in Supplementary Figure 11 c. Both the resulting "transient" and accompanying FFT spectrum show significant loss of detail, particularly for beat frequencies larger than ~250 cm$^{-1}$. This process is repeated to account for the probe pulse width, the resulting pump-probe "blurred" autocorrelation function is shown in Supplementary Figure 11 e. We note one important caveat: the "blurred" autocorrelation function will only resemble the isolated experimental transients assuming the wavepacket is projected onto a single cationic state. As discussed in Supplementary Discussion 1, this assumption is incorrect and the consequences are discussed in the main paper. Figure 12 shows the pump-probe "blurred" autocorrelation functions and calculated FFT($\Delta t$) at $\lambda_{\rm pu}$ = 348 and 344 nm. The time-autocorrelation function was not evaluated for $\lambda_{\rm pu}$ = 330 nm due to the absence of recorded LIF intensity beyond ~1200 cm$^{-1}$.

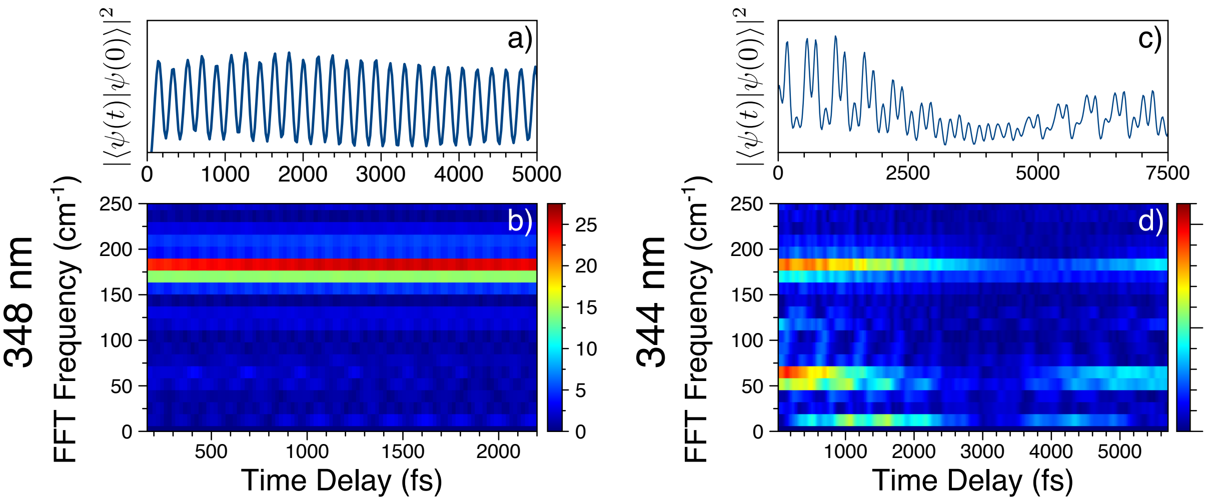


**Supplementary Figure 12:** Pump-probe "blurred" time-autocorrelation and FFT($\Delta t$) spectra at $\lambda_{\mathrm{pu}}$ = 348 nm (a and b) and 344 nm (c and d).

# Supplementary Note 1

Geometries of the $S_{0,\mathrm{eq}}$ (`S0_geom.csv`), $S_{1,\mathrm{eq}}$ (`S1_geom.csv`), and $D^{+}_{0,\mathrm{eq}}$ (`D0+_geom.csv`) states of MA used in the present work may be found in the Zenodo data repository.[10] Files contain atom labels (*e.g.* `C`) and Cartesian coordinates in four columns; distances are in Angstroms (Å).

All TR-PES transient data may be found in the Zenodo data repository.[10] Transients are formatted as comma separated variable text files: each column is the photoelectron intensity at one time delay (labeled in the first row) with one eKE value per row (labeled in the first column). Files are labeled with the molecule, pump wavelength, probe wavelength, and a date (*e.g.* `MA_344_285_18June2018.csv`).

The MATLAB code used to calculate the wavepacket "transients" is also contained in the Zenodo data repository[10] as `wavepacket.m`. The code takes user inputs such as the LIF spectral and laser pulse information listed in Supplementary Tables 3 and 4.

# Supplementary References

[1] T. Beyer and D. F. Swinehart, Commun. ACM **16**, 379 (1973).

[2] S. Lochbrunner, T. Schultz, M. Schmitt, J. P. Shaffer, M. Z. Zgierski, and A. Stolow, J. Chem. Phys. **114**, 2519 (2001).

[3] N. d. N. Rodrigues, N. C. Cole-Filipiak, M. D. Horbury, M. Staniforth, T. N. V. Karsili, Y. Peperstraete, and V. G. Stavros, J. Photochem. Photobiol., A **353**, 376 (2018).

[4] J. R. Knutson, D. G. Walbridge, and L. Brand, Biochem. **21**, 4671 (1982).

[5] I. H. van Stokkum, D. S. Larsen, and R. van Grondelle, Biochim. Biophys. Acta, Bioenerg. **1657**, 82 (2004).

[6] J. D. Young, M. Staniforth, M. J. Paterson, and V. G. Stavros, Phys. Rev. Lett. **114**, 233001 (2015).

[7] M. J. J. Vrakking, D. M. Villeneuve, and A. Stolow, Phys. Rev. A **54**, R37 (1996).

[8] V. G. Stavros and H. H. Fielding, J. Chem. Phys. **112**, 9343 (2000).

[9] A. Stolow, A. E. Bragg, and D. M. Neumark, Chem. Rev. **104**, 1719 (2004).

[10] N. d. N. Rodrigues, N. C. Cole-Filipiak, K. N. Blodgett, C. Abeysekera, T. S. Zwier, and V. G. Stavros, *Wavepacket insights into the photoprotection mechanism of the UV filter methyl anthranilate*, Zenodo Data Repository, doi:10.5281/zenodo.1472500, 2018.
